# A System of Operative Surgery, Based upon the Practice of Surgeons in the United States, and Comprising a Bibliographical Index and Historical Record of Many of Their Operations for a Period of Two Hundred Years

**Published:** 1852-07

**Authors:** 


*A System of Operative Surgery, based upon the Practice of Surgeons in the United States, and comprising a Bibliographical Index and Historical Record of many of their Operations for a period of two hundred years.* By Henry H. Smith, M. D., &c. &c. *Illustrated by numerous steel plates.* Part III. (8vo. pp. 251 and 19 plates.) Philadelphia, 1852; Lippincott, Grambo & Co.

We find in this handsome volume a very respectable continuation of Dr. Smith's Operative Surgery. The superior style of paper, type and illustration, that proved so attractive a feature of the preceding parts, will fully sustain the reputation of the work in the part just issued. Nor do the letter-press contents appear to be any less creditable to the zeal and industry of their enterprising compiler, than were those of the pioneer portions of his publication.

The twenty chapters and nineteen plates which constitute Part Third, are devoted to the operations practised on the neck and trunk. They comprise, therefore, many of the most important and interesting topics of the whole book. The first plate (29th of the series,) is occupied with the surgical anatomy of the neck. This is followed in the next (plate 30th) with a representation of instruments employed upon the œsophagus and trachea, including Bond's œsophagial forceps, Goddard's stomach pump, Physick's stomach tube, Nathan Smith's œsophagial hook, and other means and appliances of this kind. Plates 31st and 32nd respectively present views of operations performed on the trachea, the œsophagus and the larynx. Plate 33d exhibits some of the operations practised on the neck; and in plate 34th are depicted the "appearance and position of some of the tumors seen about the neck."

In plate 35th we are glad to find delineated, among other instruments, the aneurism needles of three of our Philadelphia surgeons—viz. that of Dr. Parrish, known as the Philadelphia needle, (although sometimes attributed to Dr. Mott, because that gentleman employs and recommends it), that of Dr. Horner and that of Dr. Gibson. Each of these has done good service in its time, and has been proved to be well adapted to the purpose for which it was designed.

Plates 36th and 37th present, in some twelve figures, large and small, certain operations upon the upper and lower portions of the neck, among which are included the ligature of the different arteries of those regions, and their respective relational anatomy, the strangulation of a large goitre, as effected by Liston, and the excision of the clavicle, as performed by Dr. Warren. Operations practised on the chest are well displayed in plate 38th; so likewise, in the eight following, (from 39 to 47 inclusive, chiefly copied from Bernard and Huette,) are those practised on the abdomen. These eight exhibit the opening of hepatic abscess, the treatment of wounds of the abdomen and intestines, the anatomical characteristics and relations of hernia in its different forms and conditions, together with the remedial management thereof, the operation for removal or production of artificial anus, and lastly, the ligature of the iliac arteries. The 48th plate of the series, and concluding illustration of Part Third, is occupied, in four figures, with the external characteristics of tumors of the neck and back.

So much for the engravings. We have endeavored, as briefly as possible in the foregoing enumeration, to give some idea of the nature and amount of illustration provided by the author, in connection with his two hundred and fifty-one pages of letter-press discussions and descriptions. Imperfect as the analytical sketch of these illustrations evidently is, we regret our inability to afford, in any shape whatever, a similar account of the accompanying text.

The bibliographical index, with which the part commences, exhibits, in its references to two hundred and twenty-four separate papers on the different operations and other topics, an extent of laborious research which entitles Dr. Smith to the thanks of every student of American practice. Still it is a matter of regret that, with the exception of the citations given in the incidental foot notes in the course of the work, we have the periodical literature only to refer to, and that the more accessible as well as more permanent materials collected in the comparatively small number extant of genuine American books on Surgery, have not been allowed a place at least as conspicuous as that of the more ephemeral contents of the journals of the day.

Chapter 1st is occupied with the surgical anatomy of the neck. In chapter 2d, which treats of the parotid gland and its extirpation, we are glad to note that due honor is awarded to the late Dr. Geo. M'Clellan for his share in the establishment of this operation, "having done," to use the author's language, "more than any surgeon in the United States to demonstrate its reasonable character."

Chapters 3d, 4th, 5th and 6th are devoted, in regular order of succession, to the consideration of operations on the larynx and trachea (including cauterisation of the larynx, scarification of the glottis for œdema, tracheotomy and laryngotomy); to operations on the pharynx and œsophagus; to ditto for relief of deformities of the neck, whether from torticollis or burns, and lastly, to the study and management of tumors of the neck. Then we have chapter 7th on aneurisms in general; chapter 8th on aneurism of the carotid arteries; chapter 9th on the ligature of the innominata and subclavian arteries; chapter 10th, operations upon the chest; chapter 11th, ditto on the mammary gland; chapter 12th, do. on the walls of the thorax; chapter 13th, do. upon the abdomen; and chapter 14th on wounds in the abdomen.

The remaining six chapters are pretty much occupied with the subject of hernia in its different forms, relations and results, anatomical and surgical; and with the ligature of the iliac arteries, and the removal of tumors of the back and spinal canal.

With this meagre inventory we are forced to close the present notice. Want of time unhappily prevents us from dwelling upon many matters of doctrine and detail and historical interest, that would be likely to arrest a reader's attention in the hurried perusal which alone we have been able, for the present, to afford to it. We still hope to have the opportunity, with the entire work before us, of expressing more at large, and perhaps with less indulgence, our impression of the manner in which the author has acquitted himself of some portions of his onerous task. His undertaking is certainly an arduous one, and as praiseworthy as it is difficult—one that merits all the encouragement that can honestly be given to it in its progress, and the success of which we confess ourselves unwilling to impede by any hasty words of ours. We bid him God-speed, therefore, in his labors, and take our leave of him once more with many earnest wishes for their speedy and prosperous completion.

